# Enhancement of Benzene Emissions in Special Combinations of Electronic Nicotine Delivery System Liquid Mixtures

**DOI:** 10.1021/acs.chemrestox.3c00251

**Published:** 2024-01-19

**Authors:** Fatima El Hajj Moussa, Nathalie Hayeck, Salwa Hajir, Rachel El Hage, Rola Salman, Nareg Karaoghlanian, Najat Aoun Saliba

**Affiliations:** † Department of Chemistry, Faculty of Arts and Sciences, American University of Beirut, Beirut 1107 2020, Lebanon; ‡ Center for the Study of Tobacco Products, Virginia Commonwealth University, Richmond, Virginia 23220, United States; § Mechanical Engineering Department, Maroun Semaan Faculty of Engineering and Architecture, American University of Beirut, Beirut 1107-2020, Lebanon; ∥ Department of Natural Sciences, School of Arts and Sciences, Lebanese American University, Chouran, Beirut 1102-2801, Lebanon

## Abstract

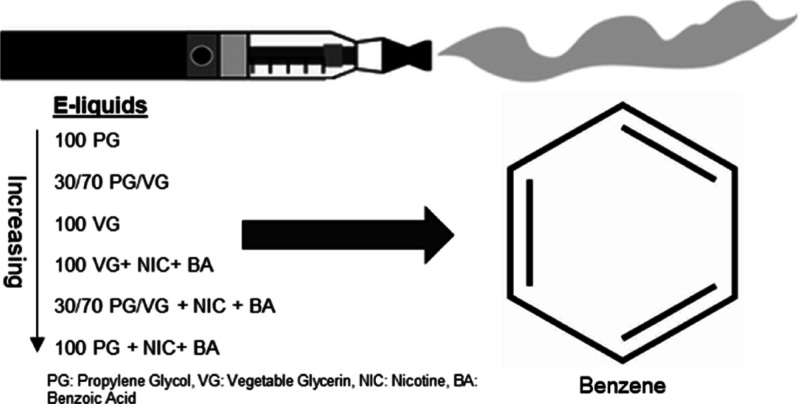

Electronic nicotine delivery systems (ENDS) are battery-powered devices introduced to the market as safer alternatives to combustible cigarettes. Upon heating the electronic liquid (e-liquid), aerosols are released, including several toxicants, such as volatile organic compounds (VOCs). Benzene has been given great attention as a major component of the VOCs group as it increases cancer risk upon inhalation. In this study, several basic e-liquids were tested for benzene emissions. The Aerosol Lab Vaping Instrument was used to generate aerosols from ENDS composed of different e-liquid combinations: vegetable glycerin (VG), propylene glycol (PG), nicotine (nic), and benzoic acid (BA). The tested mixtures included PG, PG + nic + BA, VG, VG + nic + BA, 30/70 PG/VG, and 30/70 PG/VG + nic + BA. A carboxen polydimethylsiloxane fiber for a solid-phase microextraction was placed in a gas cell to trap benzene emitted from a Sub-Ohm Minibox C device. Benzene was adsorbed on the fiber during the puffing process and for an extra 15 min until it reached equilibrium, and then it was determined using gas chromatography–mass spectrometry. Benzene was quantified in VG but not in PG or the 30/70 PG/VG mixtures. However, benzene concentration increased in all tested mixtures upon the addition of nicotine benzoate salt. Interestingly, benzene was emitted at the highest concentration when BA was added to PG. However, lower concentrations were found in the 30/70 PG/VG and VG mixtures with BA. Both VG and BA are sources of benzene. Enhanced emissions, however, are mostly noticeable when BA is mixed with PG and not VG.

## Introduction

Electronic nicotine delivery systems (ENDS) are popular devices advertised as better alternatives to traditional tobacco products.^1^ The main solvent system in the liquid reservoir is composed of vegetable glycerin (VG), propylene glycol (PG), or a mixture of VG and PG in addition to nicotine (nic). Other additives may include benzoic acid (BA) and flavoring chemicals.^2^ Due to the high demand and popularity of ENDS, public health authorities have made efforts to investigate their harmful effects on users.^3^ Specifically, several studies have compared the aerosol composition profiles of cigarettes and ENDS, which are constituted of particulate matter, metals, carbonyls, polycyclic aromatic hydrocarbons, and volatile organic compounds (VOCs).^4−6^ An ENDS device consists of a battery made of lithium that activates the device, an automizer consisting of a coil and a wick, a cartridge that holds the e-liquid, and a mouthpiece that delivers the aerosols to the user.^7^

In general, it was found that the coil resistance, type of coil, the applied power (which is directly related to the temperature), solvent constituents, and the puff topography affect the aerosol chemical composition in ENDS.^8,9^

Gas-phase aerosol constituents, especially VOCs including benzene, toluene, ethylbenzene, and xylene isomers (BTEX) are highly important due to their toxicity and carcinogenicity as defined by the International Agency for Research on Cancer (IARC).^10−12^ Being the most toxic among the BTEX group, benzene is commonly analyzed with gas chromatography coupled with mass spectrometry (GC–MS).^13^ Different methods have been reported in the literature to trap and quantify benzene from conventional cigarettes and ENDS smoke, including solvent-based impingers and solid sorbent tubes with thermal desorption.^13,14^ Other methods involved collecting aerosols in a gas sampling bag while utilizing solid-phase microextraction (SPME) for extracting the compounds of interest.^15^ Despite the robustness of the static methods presented, there is still a need to develop an in situ collection and sampling method to identify VOCs and benzene.

In the present study, the quantification of benzene emissions from e-liquids of different compositions of VG and PG was assessed by using a novel SPME–GC–MS method. Moreover, the effect of adding nic and BA on benzene emissions was also assessed.

## Methodology

### Standards, Solvents, and SPME Fiber

PG (99.5%; CAS no. 57-55-6), VG (99–101%; CAS no. 56-81-5), nic (≥99%), BA (≥99.5%), and the 85 μm SPME fiber made of carboxen polydimethylsiloxane (CAR-PDMS) were purchased from Sigma-Aldrich. An ENDS device (Sub-Ohm Minibox C), stainless steel organic cotton coils, and a nichrome coil (0.50 Ω resistance) were purchased from online vendors. A BTEX standard tank (1 ppm, Restek) was used to prepare different concentrations of benzene balanced with nitrogen gas (>99.995%) for the calibration curve. Five-liter Tedlar bags made up of polyvinyl fluoride film were purchased from Cel-Sci Corp.

### Preparation of Calibration Standards

The calibration curve was prepared using the 1 ppm BTEX gas standard. Five Tedlar bags were filled with 1 L nitrogen gas (dilution gas) by using a mass flow controller (500 mL/min). Then, using a gastight syringe, five different concentrations of benzene were prepared (2, 5, 10, 20, and 40 ppb) which are equal to 7.43, 18.55, 37.10, 74.17, and 148.34 μg/m^3^. After that, three puffs were generated from each bag using the same sampling setup as for the ENDS. The SPME fiber was exposed to the three puffs, followed by 15 min of static exposure to mimic the ENDS sampling procedure.

### SPME–GC–MS Method Validation

The repeatability and reproducibility of the SPME–GC–MS method were assessed at three different concentrations (2; 10; and 40 ppb). In addition, the calibration curve for benzene determination was generated on three different days for three consecutive weeks to ensure the reproducibility of the developed method. The limit of detection (LOD) and limit of quantification (LOQ) were obtained by quantifying the benzene concentration in 10 blank Tedlar bags filled with nitrogen gas only. The LOD is determined as equal to 3 times the standard deviation of the 10 repetitions divided by the slope of the calibration curve and the LOQ equal to 10 times the standard deviation divided by the slope.

### Optimization of the Experimental Setup

The optimization of the experimental conditions included determining the number of puffs, the power, preparation of the calibration curve, the gas cell, the SPME conditioning time, the position of the SPME fiber in the gas cell, and the SPME exposure time. The summarized parameters are listed in Table 1.

**Table 1:** Optimization of the Experimental Parameters and Setup

parameters	experimented	adopted	justification
number of puffs	3, 5, 10, and 15	3	to avoid the buildup of contamination in the ENDS device which might lead to higher values of VOCs
power (W)	15, 30, and 45	45	lower powers did not produce detectable concentrations of benzene
pressure applied by ALVIN	positive pressure and negative pressure	negative pressure	to mimic the smoker’s behavior while using ENDS
preparation of the calibration curve	(a) direct exposure of the SPME to standards prepared in a Tedlar bag (b) mimicking the experimental collection system by replacing the ENDS device with a Tedlar bag of known BTEX concentration (shown in Figure 1)	mimicking the experimental collection system by replacing the ENDS device with a Tedlar bag of known BTEX concentration (shown in Figure 1)	to compensate for any losses of VOCs on the filter/tubes during sampling
			to eliminate any changes between standards and real samples
container for gas collection	Tedlar bag and gas cell	gas cell	to allow the hybrid exposure technique (dynamic/static) of the SPME fiber
			to avoid any losses on the surface of the Tedlar bag
gas cell conditions	(a) no cleaning and not allowing the cell to cool down (b) cleaning in between sampling, allow for drying using an oven, and cool before the next use	cleaning in between sampling, allow for drying using an oven, and cool before the next use	to avoid any contamination from previous sampling
		
SPME conditioning time (min)	5, 10, and 20	5	a complete desorption of VOCs from the SPME fiber was achieved in 5 min
SPME exposure time to the gas phase	during puffing and for an additional 10,15, and 20 min	during puffing and for an additional 15 min	15 min were necessary to reach equilibrium (shown in Figure 2)

### Aerosol Generation and Benzene Sampling

Aerosols were generated using the Aerosol Lab Vaping Instrument (ALVIN).^16^ ALVIN is a digital puffing machine that replicates the puffing behavior of ENDS users. An ENDS device fitted with a coil head was used to generate aerosols at 45 W. A total of three 4 s puffs were produced with a 10 s inter-puff interval at a flow rate of 8 L/min, divided into two flows: the first passing through the sampling line, which was equal to 1 L/min, while the remaining 7 L/min were sampled through a HEPA filter to collect the generated aerosols and protect the pump. The e-liquids tested in this experiment were PG, PG + nic + BA, VG, VG + nic + BA, 30/70 PG/VG, and 30/70 PG/VG + nic + BA. Nic and BA concentrations were set at 15 and 12 mg/g, respectively. For each type of e-liquid, three replicate experiments were performed using three different coils.

**Figure 1:**
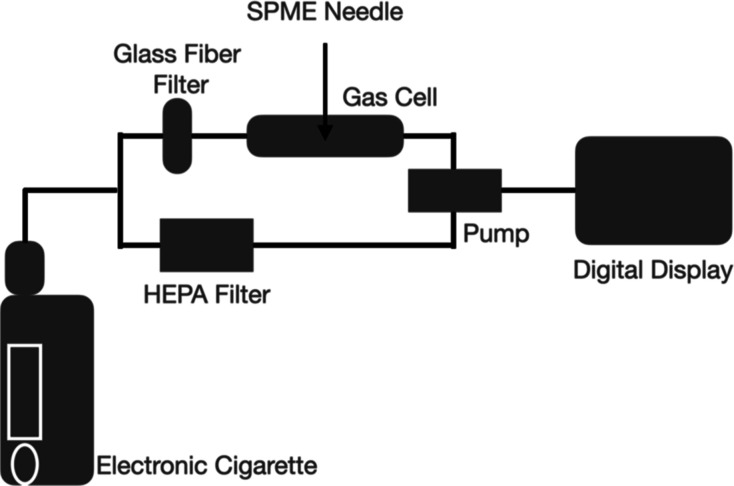
Scheme showing aerosol generation from ENDS using ALVIN.

In the sampling line, a glass fiber filter pad was placed upstream to trap particulate matter and allow passage of the gas phase into the gas cell. Benzene was collected with a CAR-PDMS SPME fiber initially placed at a fixed position in the cell. The SPME fiber was exposed to a total of three running puffs (dynamic), and then it was subjected to a total of 15 min exposure (static) until reaching equilibrium before being injected into the GC inlet. Figure 1 is an illustration of the sampling setup used to trap benzene in this experiment.

### GC–MS Parameters

For GC–MS analysis, a Thermo Scientific Trace GC Ultra System coupled with a triple quadrupole spectrometer was used, equipped with Xcalibur software. The SPME fiber was desorbed in split mode (1:25) for 1 min at 290 °C. Benzene was detected using a DB-5MS:5%-phenyl-methylpolysiloxane capillary column (30 m, 0.25 mm, 0.25 μm) with helium as a carrier gas at a constant flow rate of 1 mL/min. The temperature program was set at 40 °C and held for 1 min, increased to 50 °C at a rate of 5 °C/min, then increased to 100 °C at a rate of 15 °C/min and held for 0.5 min, and then the temperature increased at a rate of 20 °C/min until reaching 250 °C, which was held for 1 min. The total run time was 14.83 min. The mass spectrometer was operated in full scan mode (*m*/*z* range from 35 to 600). The ion source was set at 250 °C in the electron impact ionization mode (70 eV). Benzene compound was identified based on its retention time and its mass spectrum, while its quantification was performed using the *m*/*z* 78.

### Statistical Analysis

The *t*-test was employed to evaluate disparities in benzene emissions among the tested e-liquids, with *p*-values reported in the Results Section. It is essential to note that any *p*-value mentioned within the manuscript relates to the e-liquid with the highest benzene concentration compared to those of each of the other two studied e-liquids separately. A *p*-value of ≤0.05 signifies a statistically significant difference between the two sets of measurements.

## Results

### SPME–GC–MS Method Validation

**Figure 2:**
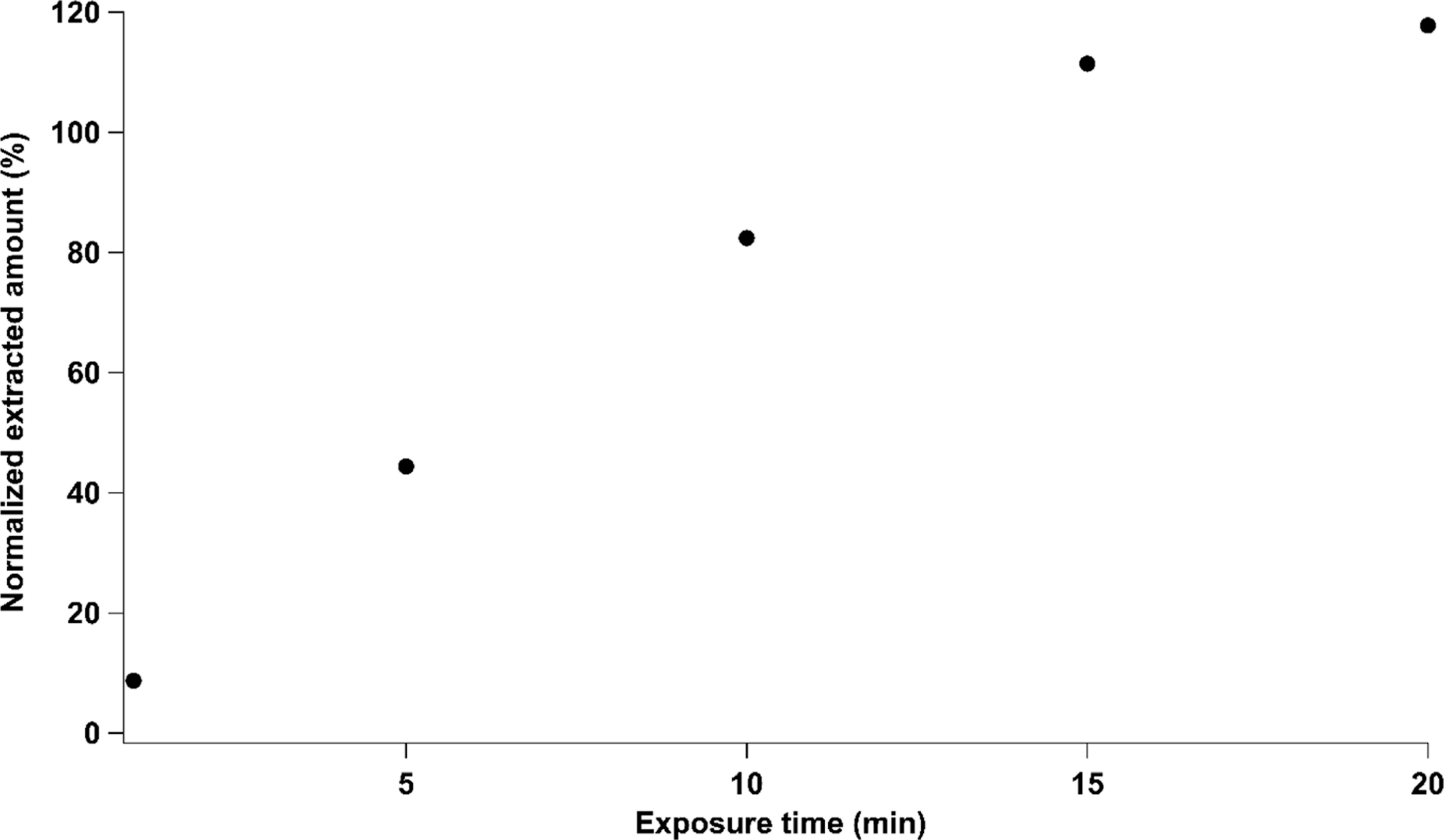
Normalized extracted benzene amount at different extraction times.

One of the most crucial parameters of an SPME extraction is the extraction time during which the fiber is exposed to the sample. As shown in Figure 2, an equilibrium between the SPME fiber and the benzene present in the gas cell is reached at 15 min, allowing the extraction of more than 95% of the benzene concentration.^17^ Therefore, an extraction time of 15 min was chosen for this application to ensure a reproducible extraction method.

**Figure 3:**
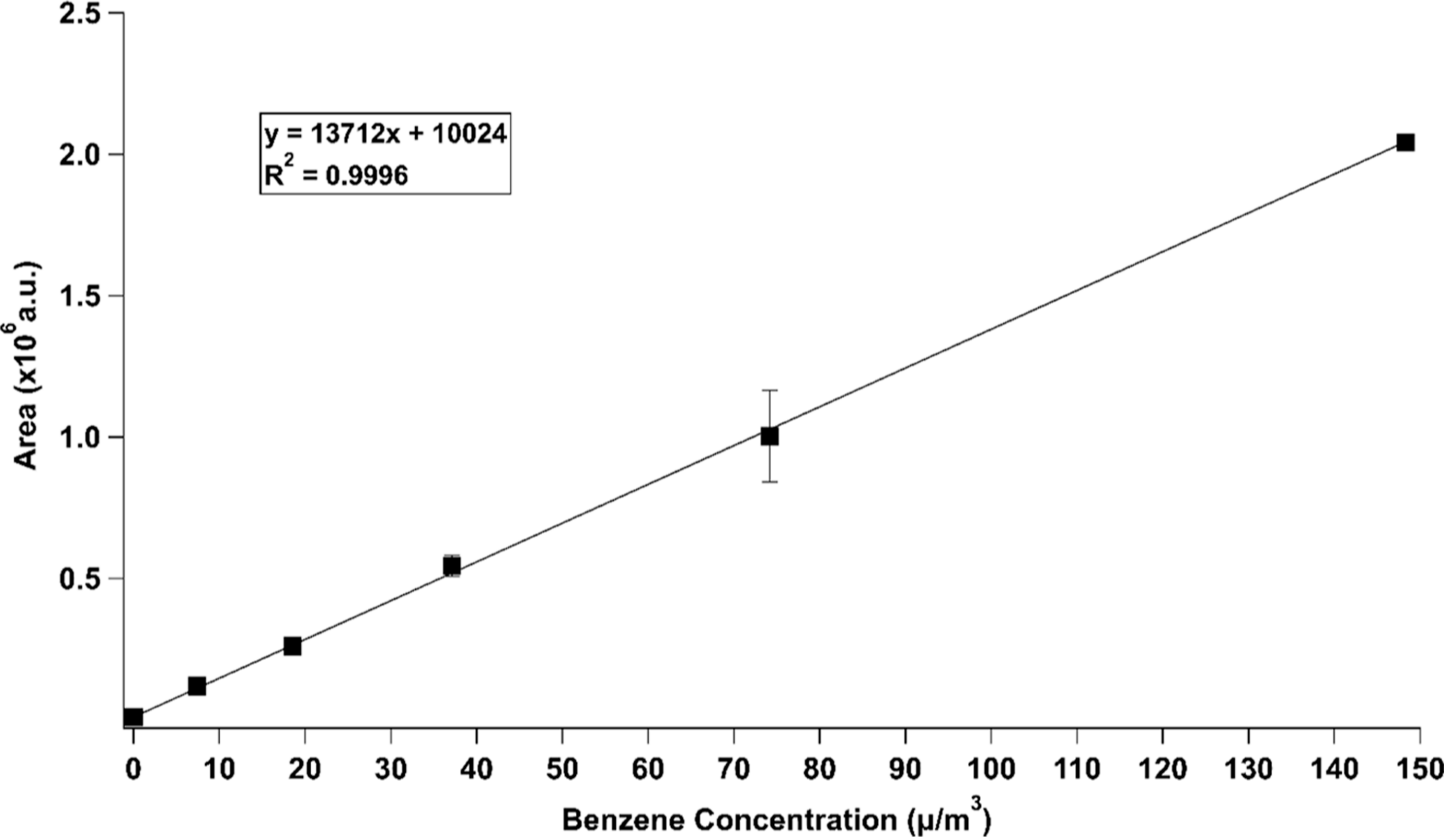
Average calibration curve obtained on three different days.

The SPME–GC–MS method presented a linearity range between 1.3 and 150 μg/m^3^ with a correlation coefficient of 0.9996 (Figure 3).

The method introduced to quantify benzene emissions from ENDS showed repeatability within an acceptable range, with relative standard deviations ranging from 2.79 to 11.45% for different concentrations. The LOD and LOQ were calculated to be 0.76 and 1.3 μg/m^3^, respectively. Table 2 facilitates a comparative analysis of the detection and quantification limits achieved by our optimized method in relation to those reported in the existing literature (units were converted from μg/m^3^ to μg/3 puffs for easier comparison). As depicted in Table 2, compared to the commonly used quantification methods, the developed SPME–GC–MS quantification method with a combination of dynamic and static exposure allows a lower LOD and LOQ for a lower number of puffs.

**Table 2:** LOD and LOQ of Benzene Reported by Different Studies and the Current Study

quantification method	LOD^a^	LOQ^b^	year	reference
SPE–GC/MS	0.15 μg/150 puffs	0.45 μg/150 puffs	2014	(13)
sampling bag-GC/MS	0.00171 μg		2012	(18)
pad/impinger-GC/MS	0.168 μg/collection	0.560 μg/collection	2016	(6)
SPME–GC/MS	0.00015 μg/3 puffs	0.00026 μg/3 puffs	2023	current study

a Limit of detection.

b Limit of quantification.

**Figure 4:**
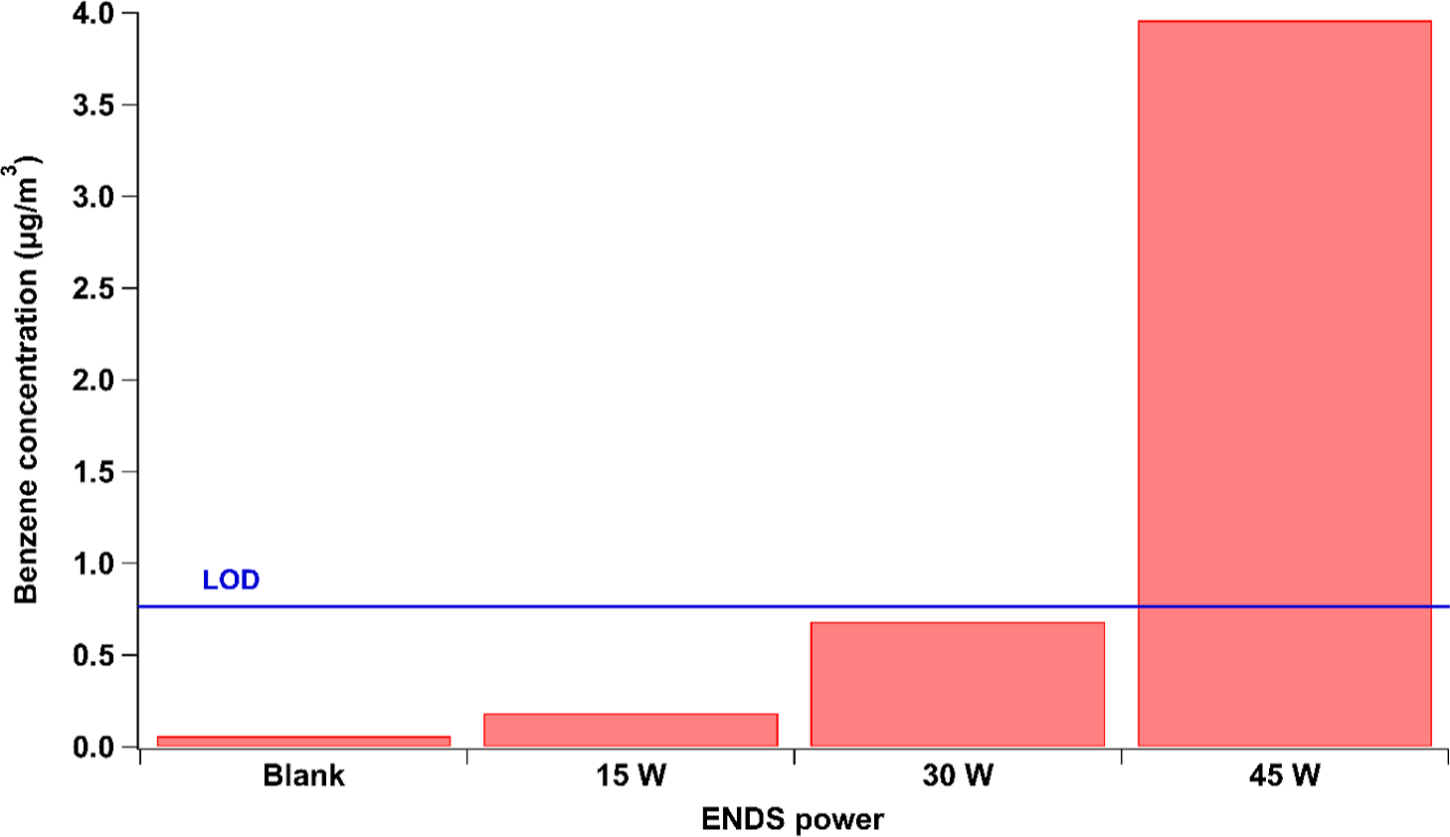
Benzene concentration generated by the ENDS under 0 W for the blank, 15, 30, and 45 W using 100% VG liquid. The blue line indicates the LOD of the SPME–GC–MS method.

To study the effect of the e-liquid composition on the emitted amount of benzene, several powers were tested using a 100% VG solution. As shown in Figure 4, the benzene concentrations formed at 15 and 30 W are below the LOD of the method. Thus, a power of 45 W was fixed in this study.

Benzene concentrations measured from the six combinations of e-liquids are reported in Table 3. In brief, quantification of benzene using the optimized method yielded the highest level of benzene (3.96 ± 0.49 μg/m^3^) in VG e-liquid when compared to the 30/70 PG/VG mixture (*p* < 0.004) and the e-liquid comprised of PG (*p* < 0.002). Levels of benzene in the PG and 30/70 PG/VG mixtures were below the detection limit.

**Table 3:** Benzene Concentrations μg/m^3^ in the Tested e-Liquids

e-liquids	PG	30/70 PG/VG	VG	PG + nic + BA	30/70 PG/VG + nic + BA	VG + nic + BA
benzene (μg/m^3^)	BDL	BDL	3.96 ± 0.49	62.85 ± 33.4	11.64 ± 0.19	6.68 ± 0.57

The emission of benzene from VG increased by 69% (6.68 ± 0.567 μg/m^3^) after the addition of a nicotine benzoate salt at 15 mg/g. This increase in emission was also observed in 30/70 PG/VG and PG mixtures, where the benzene concentration went from being undetected in the absence of nicotine benzoate salt to 11.64 ± 0.19 and 62.85 ± 33.4 μg/m^3^ in 30/70 PG/VG + nic + BA and PG + nic + BA, respectively.

## Discussion

This study aims to validate the SPME sampling method of benzene to allow the determination of its emission from ENDS. Contrary to the existing sampling methods which adopt a high number of puffs (15 and above) to quantify benzene and other VOCs, this SPME–GC–MS technique reduced the number of puffs to three. This limited number of puffs ensures the determination of real exposure of ENDS users while at the same time reducing the buildup of any VOCs during the puffing process. The high sensitivity of the SPME techniques along with the combination between a static and a dynamic sampling procedure resulted in a very low LOD (0.00015 μg/3 puffs) and LOQ (0.00026 μg/3 puffs) compared to the existing analytical methodologies used to quantify benzene such as SPE, sampling bags, and pad/impingers.

When applied to ENDS, this quantification method revealed that the VG carrier produces higher concentrations of benzene compared to PG and 30/70 PG/VG mixtures. These results align with the mechanisms proposed by Ooi et al. (2019), indicating that, unlike VG, pure PG did not produce benzene due to the absence of the “acrolein” precursor.^19^ As a result, any decrease in the percentage of VG in the e-liquid would lead to a direct decrease in the concentration of benzene. Therefore, the detected level of benzene in the tested 30/70 PG/VG e-liquid dropped below the detection limit. This outcome is comparable to the drop by a factor of 3 in the relative concentration of benzene reported by Ooi et al.^19^ when comparing 80/20 and 50/50 VG/PG e-liquids. The levels of benzene increased in PG + nic + BA, VG + nic + BA, and 30/70 PG/VG + nic + BA mixtures upon the addition of nic and BA to the tested e-liquids. This observation indicates that the decarboxylation reaction of BA serves as an additional source of benzene emissions, as previously reported in the literature.^20^ More importantly, analysis of the results showed that an increase in the PG fraction in the e-liquid, in the presence of nic and BA, leads to an increase in benzene emissions. This can be attributed to the higher volatility of PG compared to VG. According to literature reports, the vapor pressure of PG at 188 °C (the boiling point of PG at standard atmospheric pressure) is 1.01 bar, whereas that of VG is 3.32 × 10^–2^ bar.^21^ Therefore, a larger fraction of PG in the solvent mixture compared to VG enhances the volatility of the e-liquid, leading to higher emissions of aerosols.^22^ This finding is further supported by the mass consumption of PG + nic + BA e-liquid (0.24 ± 0.026 g) which was significantly higher than that of 30/70 PG/VG + nic + BA (0.17 ± 0.007 g, *p* < 0.05) and VG + nic + BA e-liquids (0.16 ± 0.013, *p* < 0.04). Furthermore, studies by Talih and co-workers demonstrated similar behavior of nic both through their mathematical model and experimental investigations. Their findings confirmed that the higher volatility of PG enables e-liquids with a higher proportion of PG to evaporate more quickly compared to VG-rich e-liquids. As a result, e-liquids with a higher PG ratio exhibit a higher nic flux.^23^ Based on this understanding, it is suggested that the evaporation of e-liquids with a higher fraction of PG, in the presence of nic and BA, leads to elevated levels of benzene in the gas phase.

In this study, a highly sensitive SPME–GC–MS method was developed to quantify benzene emissions from ENDS. Thus, only three puffs were needed for the determination of benzene. In addition, this technique has the advantage of measuring the real exposure of a user to a limited number of inhaled puffs. Consequently, the use of this technique could be extended to the determination of other VOCs emitted by any ENDS. The application of this method revealed the enhanced emission of benzene when PG fraction increases in an e-liquid containing BA and nic.

## What Does This Paper Add?

- In this study, we presented a novel and sensitive extraction procedure aimed at separating and quantifying benzene emitted by ENDS.
- At 45 W, a higher PG fraction in the BA containing e-liquid results in increased benzene formation.
